# A Zn(II) Coordination Polymer for Fluorescent Turn-Off Selective Sensing of Heavy Metal Cation and Toxic Inorganic Anions

**DOI:** 10.3390/molecules29122943

**Published:** 2024-06-20

**Authors:** Yaxin Li, Mouyi Zhang, Ying Wang, Lei Guan, Di Zhao, Xinyu Hao, Yuting Guo

**Affiliations:** School of Petrochemical Engineering, Liaoning Petrochemical University, Fushun 113001, China

**Keywords:** coordination polymer, fluorescent, selective sensing, metal cation, inorganic anion

## Abstract

A novel coordination polymer [Zn(atyha)_2_]*_n_* (**1**) (Hatyha = 2-(2-aminothiazole-4-yl)-2- hydroxyiminoacetic acid) was constructed by hydrothermal reaction of Zn^2+^ with Hatyha ligand. CP **1** exhibits a 2D (4,4)-connected topological framework with Schläfli symbol of {4^4^·6^2^}, where atyha^−^ anions serve as tridentate ligands, bridging with Zn^2+^ through carboxylate, thiazole and oxime groups. CP **1** displays a strong ligand-based photoluminescence at 390 nm in the solid state, and remains significantly structurally stable in water. Interestingly, it can be utilized as a fluorescent probe for selective and sensitive sensing of Fe^3+^, Cr_2_O_7_^2−^ and MnO_4_^−^ through the fluorescent turn-off effect with limit of detection (LOD) of 3.66 × 10^−6^, 2.38 × 10^−5^ and 2.94 × 10^−6^ M, respectively. Moreover, the efficient recyclability for detection of Fe^3+^ and Cr_2_O_7_^2−^ is better than that for MnO_4_^−^. The mechanisms of fluorescent quenching involve reversible overlap of UV-Vis absorption bands of the analytes (Fe^3+^, Cr_2_O_7_^2−^ and MnO_4_^−^) with fluorescence excitation and emission bands for CP **1**, respectively.

## 1. Introduction

Nowadays, coordination polymers (CPs), as one class of promising functional materials, have received widespread attention not only due to unique topologies, such as multiple active sites, modifiable channels and fascinating architectures, but also due to excellent performance and potential applications in various fields, such as catalyst carrier, proton conducting, sensing, magnetic and fluorescent materials, gas separation and storage [1,2,3,4,5,6,7,8]. In particular, the structural diversity and multi-functional characteristics of CPs aroused intense interest in the design and construction of chemical sensing materials [9,10,11,12]. It has been well documented that due to their fast response, high sensitivity and naked eye monitoring, fluorescent CPs can be one type of good chemical sensor for detecting analytes through fluorescence quenching, enhancement or shift [13,14,15,16]. Reasonable selection of metal nodes and organic linkers is of great significance for the construction of fluorescent CPs [17,18,19]. It was found that fluorescent CPs constructed by heterocyclic carboxylate ligands and *d*^10^ metal centers show excellent photoluminescence performance [20,21].

Recently, the rapid development of industry has led to inevitable increases in the discharge of hazardous species into the water environment, including heavy metal ions, inorganic oxo-anions and other industrial pollutants. Improper treating and failure to detect these pollutants in time may pose a threat to the ecosystem and cause various human diseases [22]. Fe^3+^ plays an important role in metabolic processes of the human body and is one of the indispensable substances in the uptake of oxygen through blood in the body and in the formation of DNA and RNA. Excess or deficiency of Fe^3+^ in the body can result in serious diseases, such as hereditary hemochromatosis and endotoxemia [23]. Inorganic oxo-anions, especially Cr_2_O_7_^2−^ and MnO_4_^−^, have been considered as highly toxic pollutants originating from industrial wastewater, and may cause skin allergies or even induce cancers [24]. Therefore, it is imperative to exploit fluorescent CPs to efficiently detect these hazardous species in aqueous media.

In this work, Hatyha ligand was selected to construct a CP based on three structural characteristics: (a) it contains Lewis basic N sites, which can coordinate with Zn(II); (b) it features carboxylate and oxime groups that may adopt various coordination modes to construct complicated topology; (c) it has N, O donors and acceptors, which can form hydrogen bonds and generate high-dimensional supramolecular structures. We have carried out the coordination reaction of atyha^−^ ligand with Zn(II) under hydrothermal condition to successfully synthesize a fluorescent CP [Zn(atyha)_2_]*_n_* (**1**). CP **1** features an infinite 2D-layered structure, displaying strong luminescence emission in solid state at room temperature. It was found that the fluorescence intensity of CP **1** could be markedly quenched by Fe^3+^, Cr_2_O_7_^2−^ and MnO_4_^−^ in the presence of interfering ions, respectively. CP **1** can behave as a chemical sensor based on the fluorescence turn-off effect, with the characteristics of selective and sensitive detection. Furthermore, it also has a relatively low LODs of 3.66 × 10^−6^, 2.38 × 10^−5^ and 2.94 × 10^−6^ M for Fe^3+^, Cr_2_O_7_^2−^ and MnO_4_^−^, respectively.

## 2. Results and Discussion

### 2.1. Crystal Structure of CP ***1***

**Figure 1:**
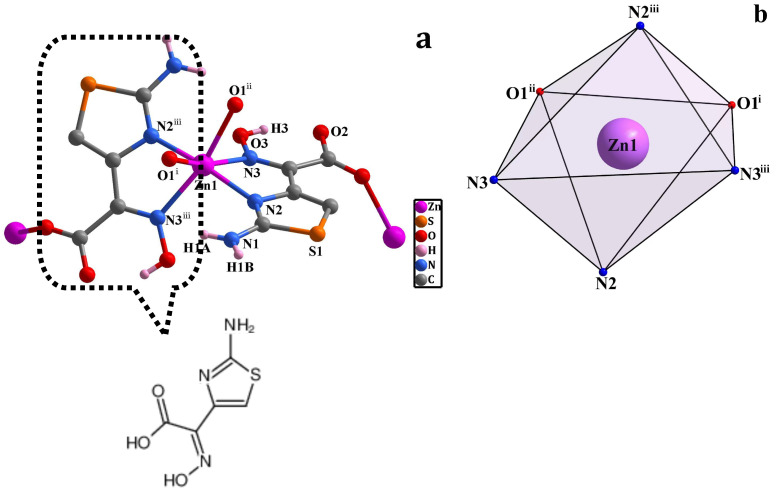

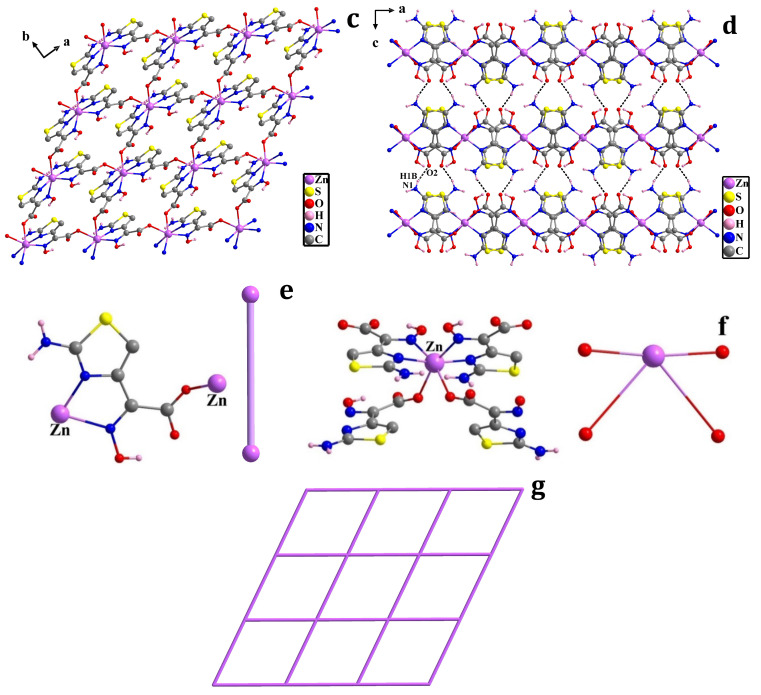
(**a**) A drawing showing the coordination environment about Zn^2+^; (**b**) octahedral coordination configuration of Zn^2+^; (**c**) 2D-layered structure; (**d**) 3D supramolecular structure; (**e**) bridged atyha^−^ ligand-based node; (**f**) 4-c node of Zn^2+^; (**g**) topological structure.

Single crystal X-ray diffraction analysis reveals that CP **1** crystallizes in the orthorhombic unit cell with space group *Pbcn* (Table 1). The asymmetric unit consists of one Zn center and two atyha^−^ ligands (Figure 1a), with the chemical formula of [Zn(atyha)_2_]*_n_*. Each Zn atom is six-coordinated by two thiazole N atoms and two oxime N atoms from two atyha^−^ ligands and two carboxylate O atoms of two atyha^−^ ligands, showing a distorted ZnN_4_O_2_ octahedral geometry (Figure 1b). The Zn-N distances are between 2.169(2) Å and 2.050(2) Å, the Zn-O lengths are 2.2504(17) Å, the O-Zn-N and N-Zn-N bond angles are in the ranges of 83.84(8)°–162.22(8)° and 76.20(9)°–169.18(13)° (Table 2), respectively, which are similar to the values reported in the references for other Zn complexes [25]. The atyha^−^ ligand adopts the carboxylate group to bind to Zn center in a monodentate coordination fashion, as well as thiazole N atom and oxime N atom chelation with Zn center, while the amino group remains uncoordinated. The atyha^−^ anions are employed as tridentate ligands to link with adjacent Zn centers, generating a 2D-layered architecture through carboxylate, thiazole and oxime groups (Figure 1c). The hydrogen bonds formed between the amino N atom and the carboxylate O atom (N1-H1B···O2^ii^) can expand the layered structure into a 3D supramolecular structure (Figure 1d). The hydrogen bonds are formed between oxime O atoms and carboxylate O atoms (O3-H3···O2), and between amino N atoms and carboxylate O atoms (N1-H1A···O1^i^ and N1-H1A···O2) (Table S1), which make the structure more stable.

From a topological perspective, each atyha^−^ ligand coordinates with two Zn centers, which can be considered as a bridging ligand-based node (Figure 1e). Each Zn center is surrounded by four atyha^−^ ligands, which can be regarded as a 4-c node (Figure 1f). Therefore, CP **1** features a 4, 4-c network structure with a Schläfli symbol of {4^4^·6^2^} (Figure 1g) [26].

### 2.2. TG Analysis

The TG analysis was performed to investigate the thermal stability of CP **1** (Figure S1). The framework of CP **1** can remain stable before the temperature reaches 196 °C. As the temperature continues to rise, the framework structure of CP **1** undergoes structural collapse and thermal decomposition at 290 °C, resulting in abrupt weight loss, and then it tends to slow down, due to the disintegration of atyha^−^ ligand. At 900 °C, CP **1** has not fully decomposed, and the TGA curve still follows a downward trend. The residue may be a mixture of ZnO and ZnS.

### 2.3. PXRD of CP **1**

The powder X-ray diffraction (PXRD) measurement was carried out to confirm the bulk phase purity of CP **1** (Figure S2). The measured pattern of CP **1** was in agreement with the simulated one generated from single crystal X-ray diffraction, revealing that the obtained bulk samples were pure phase. The sample powder of CP **1** was immersed in deionized water for 12 hours and 7 days, respectively, and after centrifugation and natural drying, the PXRD analyses were performed (Figure S3). The results revealed that the experimental patterns of the soaked samples were consistent with the simulated one, manifesting that the framework of CP **1** was intact and possessed high stability in water.

### 2.4. Fluorescence Spectrum

**Figure 2:**
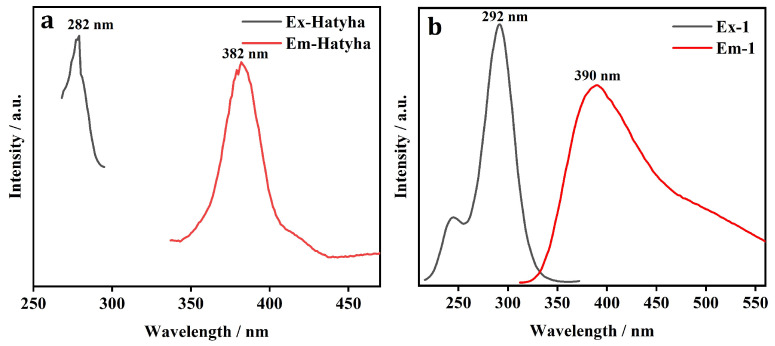
Fluorescence excitation and emission spectra of (**a**) free Hatyha ligand and (**b**) CP **1**.

The solid-state luminescent spectra of CP **1** and free Hatyha ligand were investigated at room temperature (Figure 2). The free Hatyha ligand demonstrates the maximum emission centered at 382 nm when excited at 282 nm. The band may be originated from *π*→π* and/or *n*→π* transitions [27]. The luminescent band of CP **1** displays maximum emission at 390 nm (*λ*_ex_ = 292 nm). Considering the *d*^10^ electron configuration of Zn(II), it is difficult to be oxidized or reduced. Therefore, the emission may result from neither ligand-to-metal charge transfer nor metal-to-ligand charge transfer, but the fluorescence emission of CP **1** can be attributed to intraligand *π*→π* and/or *n*→π* charge transfer [27]. In addition, the emission band of CP **1** indicates red-shift of 8 nm in comparison with those of free Hatyha ligand. This perturbation may result from the coordination interactions of atyha^−^ ligands with central metal ions.

### 2.5. Selective Sensing of Fe^3+^

**Figure 3:**
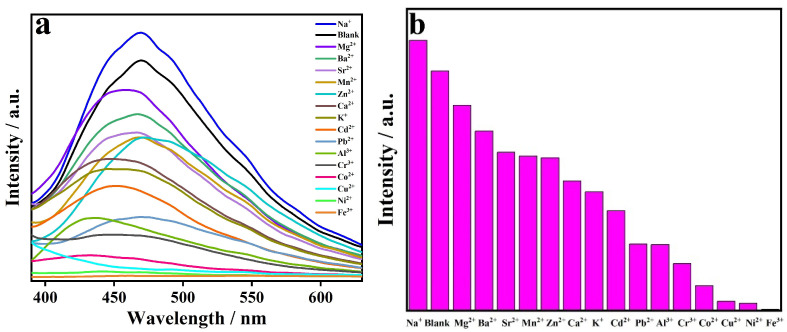
(**a**) Fluorescence spectra of CP **1** and (**b**) fluorescence intensities in different solutions of metal ions.

Considering the excellent fluorescent property and water stability of CP **1**, it can be utilized as a fluorescent sensor. From the perspective of environmental protection, water can be used as the medium; moreover, the aqueous suspension of CP **1** shows strong fluorescence intensity. Therefore, the powder sample of CP **1** was ultrasonically dispersed in water as a blank sample to investigate its fluorescent sensing behaviors toward different metal ions. Upon addition of MCl_x_ solutions (M = Na^+^, Mg^2+^, Ba^2+^, Sr^2+^, Mn^2+^, Zn^2+^, Ca^2+^, K^+^, Cd^2+^, Pb^2+^, Al^3+^, Cr^3+^, Co^2+^, Cu^2+^, Ni^2+^, Fe^3+^) to the aqueous suspensions of CP **1** (0.5 × 10^−5^ M) with metal ion concentration of 0.01 M in the mixture, the emission spectra of the suspensions were measured at the excitation wavelength of 334 nm (Figure 3a). In comparation with the blank sample, Na^+^ can cause fluorescence enhancement of CP **1;** nevertheless, the addition of other metal ions can lead to significant decreases in the fluorescence intensities of CP **1**, especially Fe^3+^ with quenching efficiency of 99.5%, indicating that CP **1** possesses efficient fluorescent turn-off sensing of Fe^3+^ (Figure 3b).

**Figure 4:**
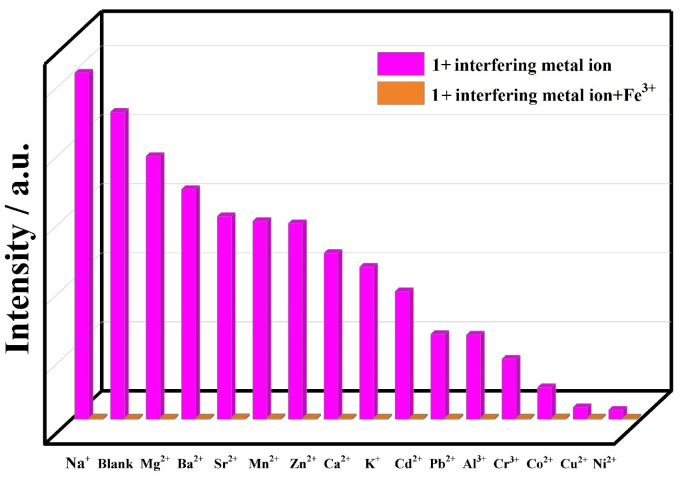
Fluorescence intensities of CP **1** in solutions with different interfering metal ions before and after addition of Fe^3+^.

In order to evaluate the anti-interference ability of CP **1** for detecting Fe^3+^, we explored the selective detection ability of CP **1** towards Fe^3+^ with competitive experiments (Figure 4), and further demonstrated that CP **1** can serve as a fluorescent turn-off sensor for detecting Fe^3+^. The fluorescence response of CP **1** towards Fe^3+^ was investigated in the presence of interfering metal ions. A 1.5 mL Fe^3+^ solution (0.01 M) was slowly dripped into a suspension of the powder sample of CP **1** with 0.01 M interfering metal ions (Na^+^, Mg^2+^, Ba^2+^, Sr^2+^, Mn^2+^, Zn^2+^, Ca^2+^, K^+^, Cd^2+^, Pb^2+^, Al^3+^, Cr^3+^, Co^2+^, Cu^2+^, and Ni^2+^), respectively. The fluorescence intensities of CP **1** decreased with a significant fluorescent turn-off effect after adding Fe^3+^. The measured result indicates that CP **1** can selectively sense Fe^3+^ without interference from other metal ions.

**Figure 5:**
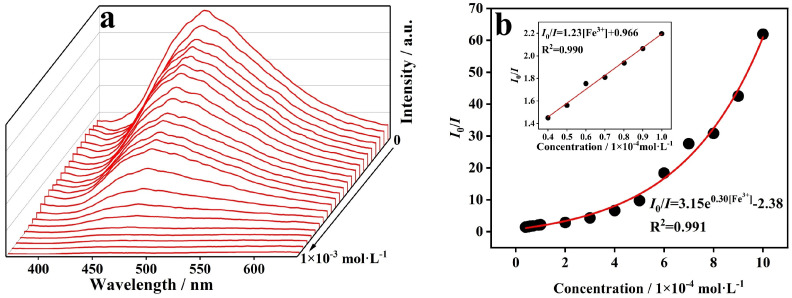
(**a**) Fluorescence responses of CP **1** in solutions with different concentrations of Fe^3+^ and (**b**) Stern–Volmer plot.

In order to further evaluate the detection sensitivity of CP **1** toward Fe^3+^ in detail, a titration experiment of quantitative fluorescence quenching was performed at the excitation wavelength of 334 nm (Figure 5a). With the addition of Fe^3+^, the fluorescence quenching efficiency sequentially increased. The relationship between the concentration of Fe^3+^ and the fluorescence intensity of the suspension of powder sample of CP **1** can be analyzed with the Stern–Volmer equation: I_0_/I = K_sv_[M] + 1 in the range of low concentration, where I_0_ and I are fluorescence intensities of the suspension of powder sample of CP **1** before and after the addition of Fe^3+^, respectively, K_sv_ is the slope of the linear curve (quenching coefficient) and [M] is the molar concentration of Fe^3+^ (Figure 5b). The result indicates that the relationship conforms to the linear equation of I_0_/I = 0.123[Fe^3+^] + 0.966 with the linear correlation (*R*_2_) of 0.990 and K_sv_ of 1.23 × 10^4^ M^−1^. Meanwhile, the LOD of CP **1** toward Fe^3+^ was further calculated to be 3.66 × 10^−6^ M by using the equation of LOD = 3σ/K_sv_, where σ is the standard deviation. We performed 11 consecutive measurements on the blank sample of CP **1** to obtain 11 fluorescence intensity values, and then calculated the σ value to be 0.015 [28].

**Figure 6:**
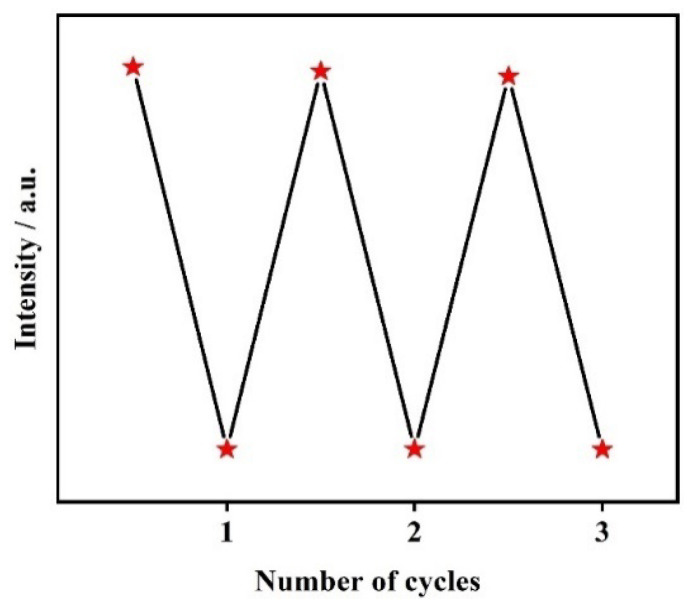
Recyclability of CP **1** for sensing of Fe^3+^.

As the recyclability of CP **1** for sensing of Fe^3+^ can increase its potential application, recycling experiments were performed (Figure 6). After the first quenching induced by Fe^3+^, it was regenerated by centrifugation and washing several times with deionized water, and its fluorescence quenching effect towards Fe^3+^ was examined again. The result revealed that the fluorescence intensity and quenching efficiency of CP **1** remained almost unchanged through at least three cycles of use, which indicates that CP **1** can be reused for detecting Fe^3+^ in water.

### 2.6. Mechanism of Fluorescence Response to Fe^3+^

**Figure 7:**
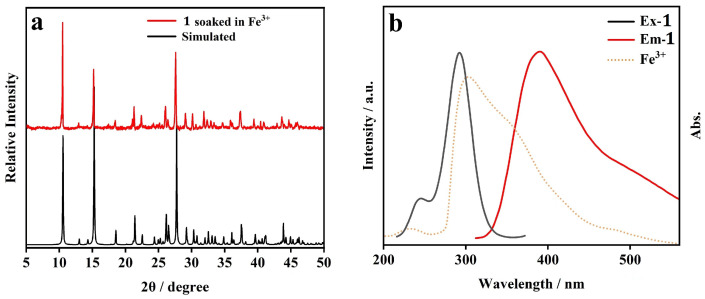
(**a**) PXRD pattern of CP **1** soaked in Fe^3+^ solution; (**b**) UV-Vis absorption spectrum of Fe^3+^, fluorescence excitation and emission spectra of CP **1**.

In order to investigate the mechanism of fluorescence quenching of CP **1** induced by Fe^3+^, further investigations were conducted. After immersion in a solution of Fe^3+^ for 12 h, XRD measurement of the sample of CP **1** was performed (Figure 7a). A similar comparison of PXRD patterns revealed that the crystalline framework of CP **1** was not destroyed. The substitution of the central metal ion of CP **1** with the added Fe^3+^ will take a long time; however, the fluorescence of CP **1** is quenched relatively quickly by Fe^3+^. Therefore, ion substitution is not the main reason for fluorescence quenching. The IR spectrum of the sample of CP **1** after immersion in Fe^3+^ solution essentially matched that of the pristine sample of CP **1**, indicating the non-coordination of N, O donors of functional groups in CP **1** with metal ions added (Figure S4). There was an overlap between the fluorescence emission band of CP **1** (λ_ex_ = 292 nm) and the UV-Vis absorption spectrum of Fe^3+^ (Figure 7b), which indicates that fluorescence resonance energy transfer occurs in the sensing process. In addition, the UV-Vis absorption spectrum of Fe^3+^ presented large overlap with the excitation band of CP **1** (λ_em_ = 390 nm) (Figure 7b), which indicated that the competitive absorption might be the main cause of fluorescence quenching of CP **1** by Fe^3+^. In summary, based on the above results, the plausible mechanism of the quenching phenomenon can be attributed to the combined effect of resonance energy transfer and competitive energy absorption [28].

### 2.7. Selective Sensing of Cr_2_O_7_^2−^ and MnO_4_^−^

**Figure 8:**
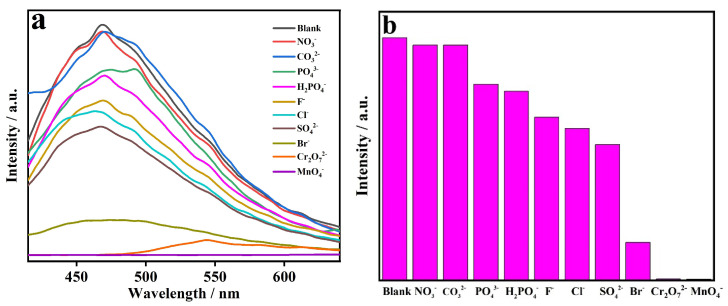
(**a**) Fluorescence spectra and (**b**) fluorescence intensities of CP **1** in different anionic solutions.

**Figure 9:**
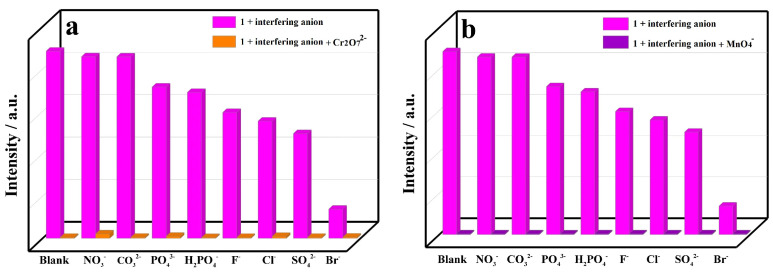
Fluorescence intensities of CP **1** in solutions with different interfering anions before and after addition of (**a**) Cr_2_O_7_^2−^ and (**b**) MnO_4_^−^.

Industrial wastewater is usually composed of coexisting metal cations and inorganic anions, and thus, its analysis poses multiple challenges. Sensing measurements were further performed at the excitation wavelength of 334 nm to explore the fluorescence responses of CP **1** to different inorganic anions (Figure 8a). A powder sample of CP **1** was ultrasonically dispersed in 0.01 M aqueous solutions of various potassium salts KM_x_ (M = NO_3_^−^, CO_3_^2−^, PO_4_^3−^, H_2_PO_4_^−^, F^−^, Cl^−^, SO_4_^2−^, Br^−^, Cr_2_O_7_^2−^ and MnO_4_^−^), generating a suspension solution, respectively. It was found that inorganic anions induced different fluorescence responses on CP **1** (Figure 8b), especially Cr_2_O_7_^2−^ and MnO_4_^−^, which could cause obvious fluorescent turn-off effects with quenching efficiencies of 99.4% and 99.5%, respectively. The results indicate that Cr_2_O_7_^2−^ and MnO_4_^−^ can be detected by CP **1** in aqueous solution. In addition, interference experiments were performed to detect Cr_2_O_7_^2−^ and MnO_4_^−^ in the presence of other inorganic anions, respectively (Figure 9). The fluorescence quenching intensity of CP **1** towards Cr_2_O_7_^2−^ and MnO_4_^−^ remained constant as the interfering anion was changed, respectively. It was evident that the fluorescent turn-off effects of Cr_2_O_7_^2−^ and MnO_4_^−^ on CP **1** were almost unaffected by the interfering inorganic anions, respectively.

**Figure 10:**
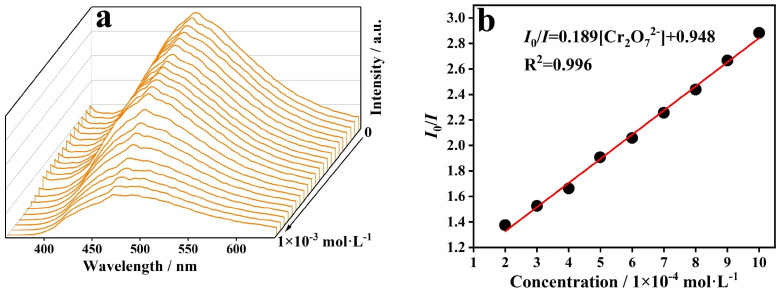
(**a**) Fluorescence responses of CP **1** in solutions with different concentrations of Cr_2_O_7_^2−^ and (**b**) Stern–Volmer plot.

**Figure 11:**
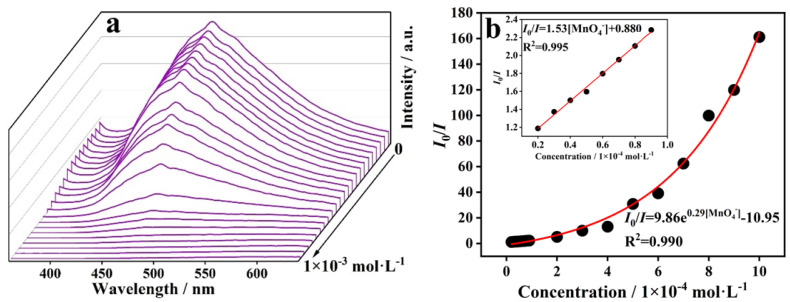
(**a**) Fluorescence responses of CP **1** in solutions with different concentrations of MnO_4_^−^ and (**b**) Stern–Volmer plot.

A titration experiment showed that the fluorescence intensity of CP **1** was gradually quenched at the excitation wavelength of 334 nm with the addition of Cr_2_O_7_^2−^ and MnO_4_^−^, respectively (Figure 10 and Figure 11). Moreover, the K_sv_ curves of Cr_2_O_7_^2−^ and MnO_4_^−^ showed good linear correlation, while the K_sv_ values for Cr_2_O_7_^2−^ and MnO_4_^−^ were 1.89 × 10^3^ M^−1^ and 1.53 × 10^4^ M^−1^, respectively. Meanwhile, the LODs of Cr_2_O_7_^2−^ and MnO_4_^−^ were 2.38 × 10^−5^ and 2.94 × 10^−6^ M, respectively. The experimental results indicate that CP **1** can sensitively detect Cr_2_O_7_^2−^ and MnO_4_^−^ in water.

**Figure 12:**
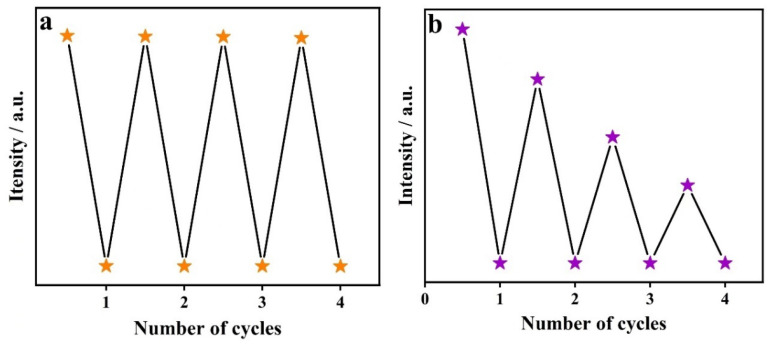
Cyclic experiments using CP **1** for detection of (**a**) Cr_2_O_7_^2−^ and (**b**) MnO_4_^−^.

To evaluate the recyclability of CP **1** as a fluorescent sensor, the fluorescence of CP **1** was repeatedly quenched by Cr_2_O_7_^2−^ and MnO_4_^−^, respectively (Figure 12). After each quenching, the powder sample of CP **1** was recovered through centrifugation, water washing and drying. The experimental results indicated that the fluorescent intensity of CP **1** can almost be restored after exposure to Cr_2_O_7_^2−^ through at least four cycles; furthermore, the fluorescence quenching efficiency also remains unchanged, indicating that CP **1** has good recyclability for detecting Cr_2_O_7_^2−^. However, the fluorescent intensity of CP **1** could not be restored for sensing of MnO_4_^−^, revealing its poor reversibility.

### 2.8. Mechanism of Fluorescence Response to Cr_2_O_7_^2−^/MnO_4_^−^

**Figure 13:**
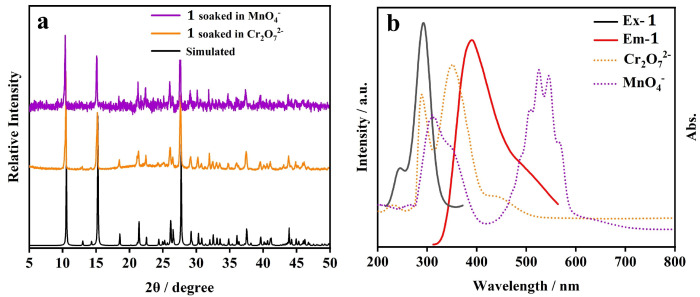
(**a**) PXRD patterns of CP **1** soaked in Cr_2_O_7_^2−^ and MnO_4_^−^ solution, respectively; (**b**) UV-Vis absorption spectra of Cr_2_O_7_^2−^ and MnO_4_^−^, fluorescence excitation and emission spectra of CP **1**.

In order to elucidate the mechanism of the fluorescence quenching of CP **1** induced by Cr_2_O_7_^2−^/MnO_4_^−^, additional measurements were performed. The PXRD pattern of a sample of CP **1** immersed in the solution of Cr_2_O_7_^2−^/MnO_4_^−^ was consistent with the simulated one (Figure 13a), indicating that the fluorescence quenching was not caused by the collapse of the structure. The IR spectrum of the sample of CP **1** treated by the solution of Cr_2_O_7_^2−^/MnO_4_^−^ essentially matched with that of the pristine sample, indicating that there was no weak interaction between CP **1** and the inorganic anions added (Figure S4). In addition, the UV-Vis absorption spectra of Cr_2_O_7_^2−^/MnO_4_^−^ overlapped the emission band of CP **1** (λ_ex_ = 292 nm) (Figure 13b), which indicated that the fluorescence quenching caused by Cr_2_O_7_^2−^/MnO_4_^−^ could be attributed to the resonance energy transfer. Moreover, a partial overlap existed between the absorption spectra of Cr_2_O_7_^2−^/MnO_4_^−^ and the excitation band of CP **1** (λ_em_ = 390 nm) (Figure 13b), which hindered the absorption of CP **1** and caused photoluminescence attenuation. Therefore, there is clear evidence for the competitive adsorption between each analyte and CP **1**. In summary, the fluorescence quenching mechanism of Cr_2_O_7_^2−^/MnO_4_^−^ on CP **1** is mainly attributed to the resonance energy transfer, as well as the competitive energy absorption [28].

### 2.9. Comparison with Other, Related Sensors

Zn(II) CPs have been proven to be excellent sensing materials for metal cations and inorganic anions, etc. Some of them can only be utilized for the detection of a single analyte. For instance, a 2D Zn(II) CP [Zn(4-PP)(1,4-BDC)∙(H_2_O)]_n_ (1,4-PP = 4-(1H-pyrazol-3-yl)pyridine, 1,4-H_2_BDC = 1,4-benzenedicarboxylic acid) synthesized under hydrothermal conditions exhibits selective and sensitive detection of Fe^3+^ in water medium [29]. However, the title Zn(II) CP displays multi-functional fluorescence responses towards Fe^3+^, Cr_2_O_7_^2−^ and MnO_4_^−^. The title CP was constructed by atyha^−^ anions and Zn cations, and shows excellent sensing performances towards Fe^3+^, Cr_2_O_7_^2−^ and MnO_4_^−^, which are mainly due to the fluorescent turn-off effect. According to relevant reports in the literatures, it can be seen that these are comparable to or even better than those of other Zn(II) CPs [30,31,32,33,34]. Zhang et al. designed and prepared a Zn(II) CP as a dual-responsive luminescent sensor for Fe^3+^ and MnO_4_^−^ in water, with LODs of 5.0 × 10^−6^ M and 8.86 × 10^−6^ M, respectively [30]. Another Zn-MOF with a 1D chain structure was confirmed as a multi-functional luminescence sensor for the detection of Fe^3+^ and Cr_2_O_7_^2−^ in water through fluorescent quenching, and the LODs were 1.172 × 10^−5^ M and 2.465 × 10^−4^ M, respectively [31].

## 3. Experimental Section

### 3.1. Materials and Methods

All reagents and solvents were of reagent grade and purchased from Shanghai Aladdin Biochemical Technology Co., Ltd. Elemental analysis was performed with a Perkin-Elmer 240CHN analyzer (Perkin-Elmer Corporation, Norwalk CT, USA). FT-infrared spectra were collected by a Magna FT-IR 750 spectrometer (Nicolet, Tokyo, Japan) in the range of 4000 cm^−1^–400 cm^−1^ (KBr pellet). Thermogravimetric data were collected on a NETZSCH STA 449C (Netzsch Corporation, Bavaria, Germany) unit between 25 °C and 900 °C with a heating rate of 10 °C·min^−1^ under a nitrogen atmosphere, with an Al_2_O_3_ crucible used to hold the solid sample. Fluorescence excitation and emission spectra were measured by a Perkin-Elmer LS55 fluorescence spectrophotometer (Perkin-Elmer Corporation, Norwalk, CT, USA) using a 75W Xenon arc-lamp and an R928 photomultiplier tube as a detector for solid samples. Emission intensity measurements were carried out using the adapter and holder supplied by the manufacturer of thespectrophotometer. Excitation and emission spectra were corrected for the instrumental response. UV-Vis absorption spectra were measured by a UV-1800 spectrophotometer (ShiMadzu Corporation, Kyoto, Japan). Powder X-ray diffraction (PXRD) patterns were recorded on a Bruker D8 Advanced XRD diffractometer (Bruker, Rheinstetten, Germany) with Cu-K_α_ monochromator.

### 3.2. Synthesis of CP **1**

A mixture of Hatyha ligand (0.037 g, 0.2 mmol) and ZnCl_2_ (0.014 g, 0.1 mmol) in deionized water (20 mL), with its pH value adjusted to about 5 with NaOH (0.1 M) solution, was transferred to a 25 mL Teflon-lined autoclave and heated at 120 °C for 48 h. After cooling to room temperature, colorless blocked crystals of **1** were collected with a yield of 58% based on Zn(II). Elemental analysis (%) found: C, 27.46; H, 1.92; N, 19.26. Calcd for C_10_H_8_N_6_O_6_S_2_Zn: C, 27.42; H, 1.83; N, 19.19%. IR (KBr, cm^−1^): 3414, 3269 (ν_NH2_), 3124(ν_O-H_), 1632, 1347(ν_COO_^−^), 1512, 1424(ν_C=C, C=N_), 1194, 1083, 1028(ν_C-O_), 800(δ_C-H_), 726, 641(ν_C-S_), 535(ν_M-N_) (Figure S4).

### 3.3. X-ray Crystallography

Single-crystal X-ray diffraction data for CP **1** were recorded using a Bruker D8 VENTURE diffractometer with graphite-monochromatized Mo-K_α_ radiation (λ = 0.71073 Å) by *φ-ω* scan mode. The structure was solved by direct methods and refined by full-matrix least-squares techniques using the SHELXS and SHELXL programs, respectively [35,36]. For CP **1**, crystallographic data and structure refinement details are summarized in Table 1. Selected bond lengths and bond angles are given in Table 2.

## 4. Conclusions

In conclusion, a novel 2D Zn-based coordination polymer was hydrothermally synthesized by introducing Hatyha ligand. The atyha^−^ linkers adopted the carboxylate, thiazole and oxime groups to bridge with Zn^2+^, generating a 2D framework with Schläfli symbol {4^4^·6^2^} topology. Notably, CP **1** displays strong fluorescence emission, which is derived from intraligand transition. It exhibits high structural stabilities towards aqueous solutions. It was found that CP **1** could not only detect Fe^3+^ and Cr_2_O_7_^2−^ with high selectivity, sensitivity and recyclability, but also serve as an excellent candidate for the selective and sensitive sensing of MnO_4_^−^, indicating low LODs of 3.66 × 10^−6^, 2.38 × 10^−5^ and 2.94 × 10^−6^ M, respectively. The mechanism of the fluorescence turn-off sensing can be attributed to the synergistic effect of resonance energy transfer and competitive energy absorption.

## Figures and Tables

**Table 1:** Crystallographic data and structure refinement details for CP **1**.

	1
Chemical formula	C_10_H_8_N_6_O_6_S_2_Zn
*M*_r_	437.71
Crystal system,	Orthorhombic
Space group	*Pbcn*
Temperature(K)	253
*a*, *b*, *c* (Å)	11.6451(16), 7.2951(8), 16.761(2)
*Z*	4
*V* (Å^3^)	1423.9(3)
*µ* (mm^−1^)	2.07
No. of measured, independent and obverted [*I* > 2*σ*(*I*)] reflection	32,248, 1631, 1500
*R*_int_	0.057
(sin*θ*/*λ*)_max_ (Å^−1^)	0.650
*R*[*F*^2^ > 2σ(*F*^2^)], *ωR*(*F*^2^), *S*	0.035, 0.098, 1.08
No. of reflections	1631
No. of parameters	115
No. of restraints	1
*R*_1_, *ωR*_2_ [*I* ≥ 2*σ*(*I*)]	0.0349, 0.0956
*R*_1_, *ωR*_2_ (all data)	0.0378, 0.0978
Δ*ρ*_max_, Δ*ρ*_min_ (e·Å^−3^)	1.63, −1.37

**Table 2:** Selected bond lengths (Å) and angles (°) for CP **1**.

**Atom1-Atom2**	**Distance**	**Atom1-Atom2**	**Distance**	**Atom1-Atom2**	**Distance**
Zn1-O1^i^	2.2504(17)	Zn1-N2	2.050(2)	Zn1-N3	2.169(2)
Zn1-O1^ii^	2.2504(17)	Zn1-N2^iii^	2.050(2)	Zn1-N3^iii^	2.169(2)
**Atom1-Atom2-Atom3**	**Angle**	**Atom1-Atom2-Atom3**	**Angle**	**Atom1-Atom2-Atom3**	**Angle**
O1^i^-Zn1-O1^ii^	79.32(7)	N2^iii^-Zn1-N2	169.18(13)	N3^iii^-Zn1-O1^ii^	162.22(8)
N2-Zn1-O1^ii^	90.24(8)	N2-Zn1-N3	76.20(9)	N3-Zn1-O1^i^	162.22(8)
N2^iii^-Zn1-O1^i^	90.24(8)	N2^iii^-Zn1-N3	97.77(9)	N3-Zn1-O1^ii^	83.84(8)
N2^iii^-Zn1-O1^ii^	98.10(8)	N2^iii^-Zn1-N3^iii^	76.20(9)	N3^iii^-Zn1-O1i	83.84(8)
N2-Zn1-O1^i^	98.10(8)	N2-Zn1-N3^iii^	97.77(9)	N3^iii^-Zn1-N3	113.44(14)

Symmetry codes: (i) x+1/2, y+1/2, −z+3/2; (ii) − x+1/2, y+1/2, z; (iii) −x+1, y, −z+3/2.

## Data Availability

The data presented in this study are available on request from the corresponding author.

## Supplementary Materials

The following supporting information can be downloaded at: https://www.mdpi.com/article/10.3390/molecules29122943/s1, Figure S1: TGA curve of CP **1**; Figure S2: Measured and simulated PXRD patterns of CP **1**; Figure S3: PXRD patterns of CP **1** soaked in water for 12 h and 7 d; Figure S4: IR spectra of sample of CP **1**, as well as after immersion in Fe^3+^, Cr_2_O_7_^2−^ and MnO_4_^−^ solutions, Table S1: Hydrogen bond lengths (Å) and bond angles (°) for CP **1**.
