# Supplementary material for: A Zn(II) Coordination Polymer for Fluorescent Turn-Off Selective Sensing of Heavy Metal Cation and Toxic Inorganic Anions
Source: Molecules. 2024 Jun 20;29(12):2943. doi: 10.3390/molecules29122943 (PMC11206703; doi:10.3390/molecules29122943)

checkCIF/PLATON report

You have not supplied any structure factors. As a result the full set of tests cannot be run.

THIS REPORT IS FOR GUIDANCE ONLY. IF USED AS PART OF A REVIEW PROCEDURE FOR PUBLICATION, IT SHOULD NOT REPLACE THE EXPERTISE OF AN EXPERIENCED CRYSTALLOGRAPHIC REFEREE.

No syntax errors found. CIF dictionary Interpreting this report

Datablock: 1

```
Bond precision:   C-C = 0.0037 A          Wavelength=0.71073

Cell:               a=11.6451(16)       b=7.2951(8)      c=16.761(2)
                    alpha=90            beta=90          gamma=90
Temperature:        253 K

                Calculated                  Reported
Volume          1423.9(3)                   1423.9(3)
Space group     P b c n                     P b c n
Hall group      -P 2n 2ab                   -P 2n 2ab
Moiety formula  C10 H8 N6 O6 S2 Zn          C10 H8 N6 O6 S2 Zn
Sum formula     C10 H8 N6 O6 S2 Zn          C10 H8 N6 O6 S2 Zn
Mr              437.73                      437.71
Dx,g cm-3       2.042                       2.042
Z               4                           4
Mu (mm-1)       2.067                       2.067
F000            880.0                       880.0
F000'           882.46
h,k,lmax        15,9,21                     15,9,21
Nref            1634                        1631
Tmin,Tmax       0.764,0.813                 0.657,0.746
Tmin'           0.764

Correction method= # Reported T Limits: Tmin=0.657 Tmax=0.746
AbsCorr = MULTI-SCAN

Data completeness= 0.998           Theta(max)= 27.509

                                                     wR2(reflections)=
R(reflections)= 0.0349( 1500)                        0.0978( 1631)

S = 1.081                    Npar= 115
```

The following ALERTS were generated. Each ALERT has the format
test-name_ALERT_alert-type_alert-level.
Click on the hyperlinks for more details of the test.

Alert level C

```
PLAT430_ALERT_2_C Short Inter D...A Contact  O1       ..O1       .       2.87 Ang.
                                              -x,y,3/2-z  =      3_556 Check
```

Alert level G

```
PLAT002_ALERT_2_G Number of Distance or Angle Restraints on AtSite          1 Note
PLAT004_ALERT_5_G Polymeric Structure Found with Maximum Dimension          2 Info
PLAT007_ALERT_5_G Number of Unrefined Donor-H Atoms .............           3 Report
              H1A   H1B   H3
PLAT012_ALERT_1_G N.O.K.   _shelx_res_checksum Found in CIF ......     Please Check
PLAT172_ALERT_4_G The CIF-Embedded .res File Contains DFIX Records          1 Report
PLAT794_ALERT_5_G Tentative Bond Valency for Zn1       (II)       .       1.87 Info
PLAT860_ALERT_3_G Number of Least-Squares Restraints ............           1 Note
PLAT883_ALERT_1_G No Info/Value for _atom_sites_solution_primary .     Please Do !
```

0 **ALERT level A** = Most likely a serious problem - resolve or explain
0 **ALERT level B** = A potentially serious problem, consider carefully
1 **ALERT level C** = Check. Ensure it is not caused by an omission or oversight
8 **ALERT level G** = General information/check it is not something unexpected

2 ALERT type 1 CIF construction/syntax error, inconsistent or missing data
2 ALERT type 2 Indicator that the structure model may be wrong or deficient
1 ALERT type 3 Indicator that the structure quality may be low
1 ALERT type 4 Improvement, methodology, query or suggestion
3 ALERT type 5 Informative message, check

It is advisable to attempt to resolve as many as possible of the alerts in all categories. Often the minor alerts point to easily fixed oversights, errors and omissions in your CIF or refinement strategy, so attention to these fine details can be worthwhile. In order to resolve some of the more serious problems it may be necessary to carry out additional measurements or structure refinements. However, the purpose of your study may justify the reported deviations and the more serious of these should normally be commented upon in the discussion or experimental section of a paper or in the "special_details" fields of the CIF. checkCIF was carefully designed to identify outliers and unusual parameters, but every test has its limitations and alerts that are not important in a particular case may appear. Conversely, the absence of alerts does not guarantee there are no aspects of the results needing attention. It is up to the individual to critically assess their own results and, if necessary, seek expert advice.

Publication of your CIF in IUCr journals

A basic structural check has been run on your CIF. These basic checks will be run on all CIFs submitted for publication in IUCr journals (*Acta Crystallographica*, *Journal of Applied Crystallography*, *Journal of Synchrotron Radiation*); however, if you intend to submit to *Acta Crystallographica Section C* or *E* or *IUCrData*, you should make sure that full publication checks are run on the final version of your CIF prior to submission.

Publication of your CIF in other journals

Please refer to the *Notes for Authors* of the relevant journal for any special instructions relating to CIF submission.

PLATON version of 06/01/2024; check.def file version of 05/01/2024

Datablock 1 - ellipsoid plot

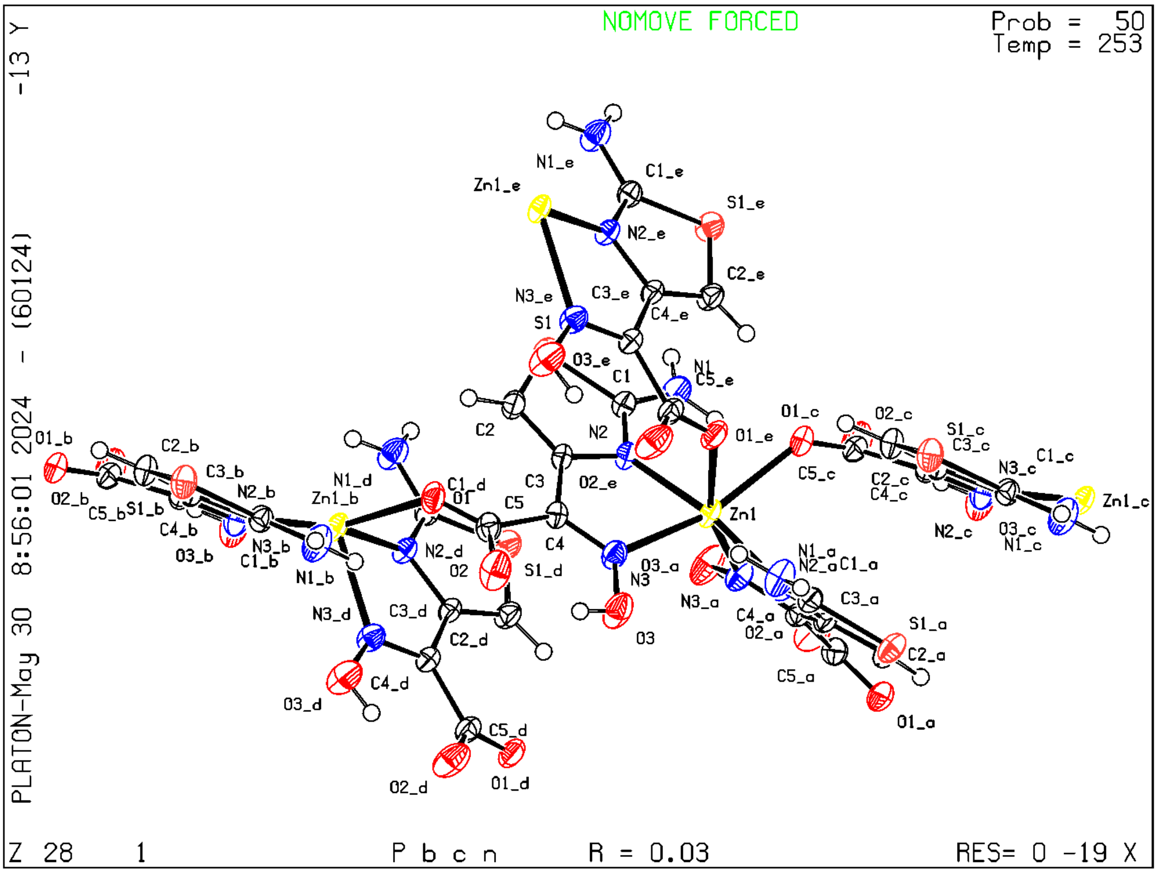

Supplement: Supplementary file 1 [file molecules-29-02943-s001.zip › checkcif.pdf]
