# Supplementary material for: A Zn(II) Coordination Polymer for Fluorescent Turn-Off Selective Sensing of Heavy Metal Cation and Toxic Inorganic Anions
Source: Molecules. 2024 Jun 20;29(12):2943. doi: 10.3390/molecules29122943 (PMC11206703; doi:10.3390/molecules29122943)
Supplement: Supplementary file 1 [file molecules-29-02943-s001.zip › molecules-3055888-Supplementary.pdf]

Supplementary Materials

TableS1. Hydrogen bond lengths (Å) and bond angles (°) for CP **1**.

D-H···A	*d*(D-H)	*d*(H···A)	*d*(D···A)	∠DHA
O3-H3···O2	0.830	1.767	2.056	147.34
N1-H1A···O1^{i}	0.870	2.595	3.183	125.85
N1-H1A···O2	0.870	2.646	3.449	154.00
N1-H1B···O2^{ii}	0.870	2.089	2.927	161.28

Symmetry codes: (i) x+1/2, y+1/2, −z+3/2; (ii) −x+1/2, −y+3/2, z−1/2.

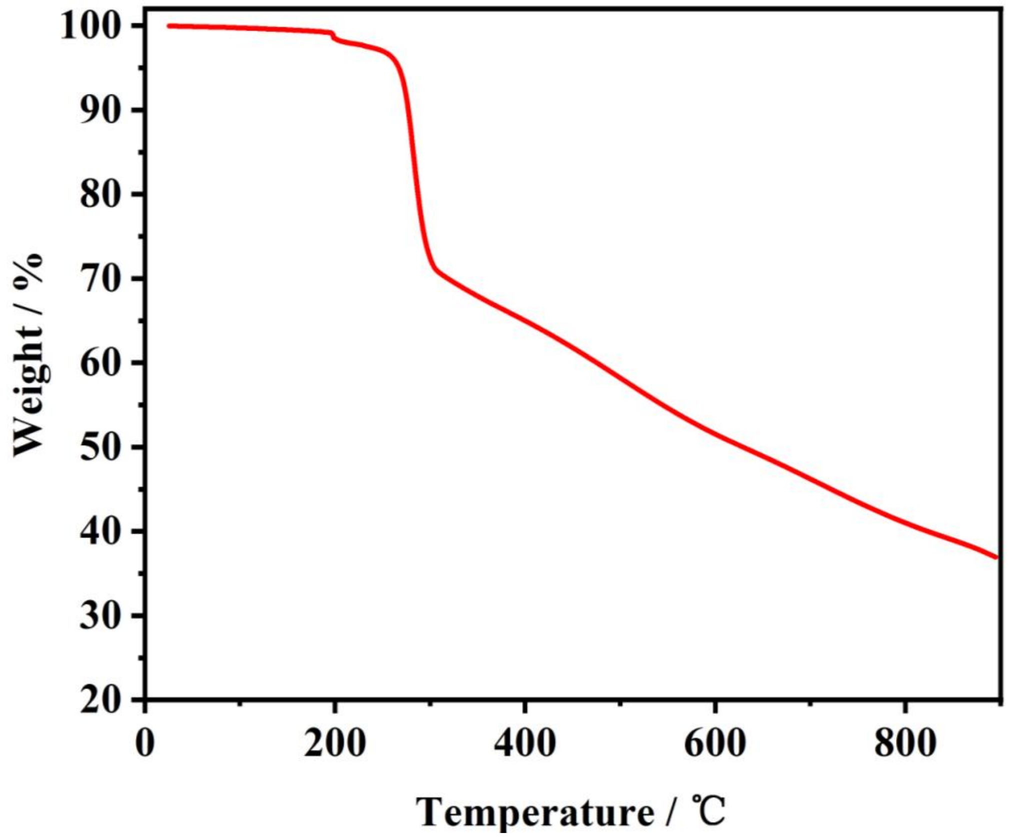

Figure S1. TGA curve of CP **1**.

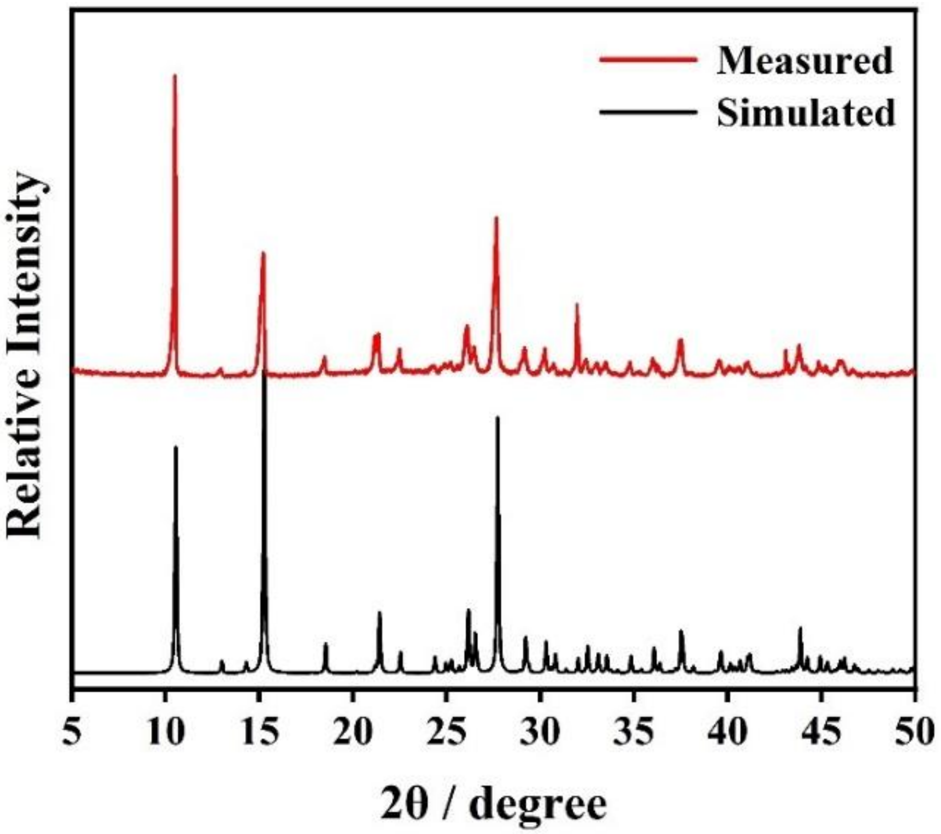

Figure S2. Measured and simulated PXRD patterns of CP **1**.

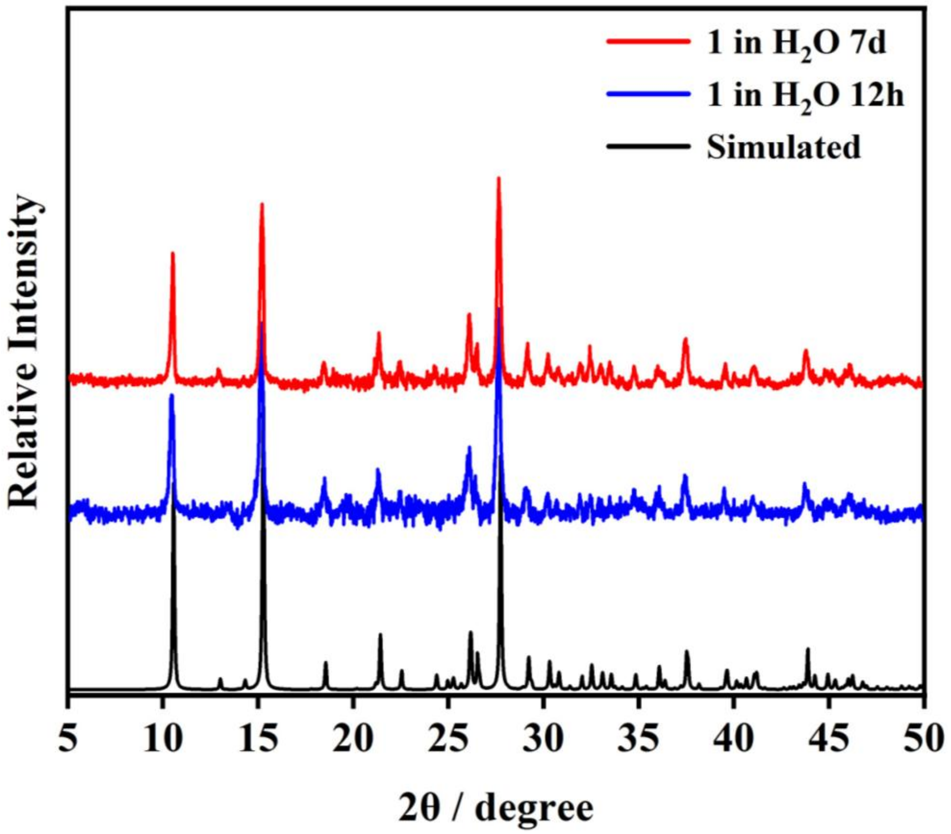

Figure S3. PXRD patterns of CP **1** soaked in water for 12 h and 7 d.

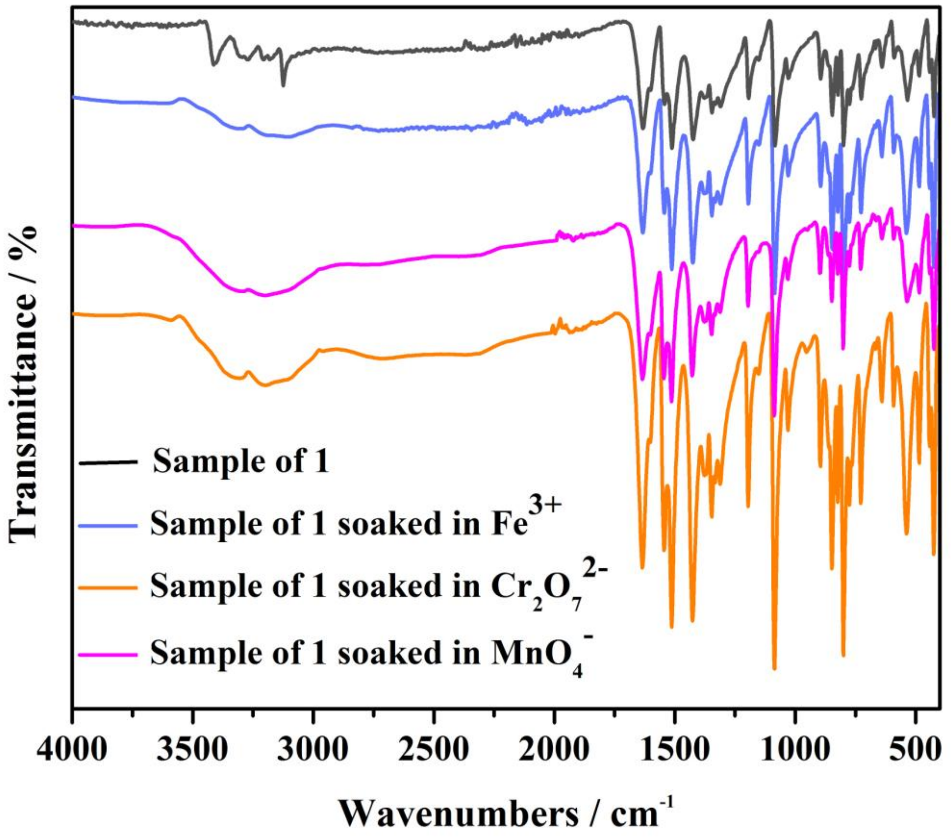

Figure S4. IR spectra of sample of CP **1**, as well as after immersion in Fe^{3+}, $Cr_2O_7^{2-}$ and MnO_4^- solutions.
